# Association of plaque characteristics with long-term stroke recurrence in patients with intracranial atherosclerotic disease: a 3D high-resolution MRI-based cohort study

**DOI:** 10.1007/s00330-023-10278-y

**Published:** 2023-10-23

**Authors:** Yaodong Lv, Xiaotong Ma, Weihua Zhao, Jiachen Ju, Peng Yan, Shan Li, Yuan Xue, YanLing Sui, Sai Shao, Qinjian Sun, Chengxuan Qiu

**Affiliations:** 1 Department of Neurology, Shandong Provincial Hospital, Shandong University, Jinan, Shandong China; 2 Department of Neurology, The Affiliated Yantai Yuhuangding Hospital of Qingdao University, Yantai, Shandong China; 3 Key Laboratory of Endocrine Glucose & Lipids Metabolism and Brain Aging, Ministry of Education; Department of Neurology, Shandong Provincial Hospital Affiliated to Shandong First Medical University, Jinan, 250021 Shandong China; 4 Department of Radiology, Shandong Provincial Hospital Affiliated to Shandong First Medical University, Jinan, 250021 Shandong China; 5 Aging Research Center, Department of Neurobiology, Care Sciences and Society, Karolinska Institutet-Stockholm University, Stockholm, Sweden

**Keywords:** Intracranial atherosclerotic disease, Plaque characteristics, Stroke recurrence, Magnetic resonance vessel wall imaging

## Abstract

**Objectives:**

To evaluate the predictive ability of plaque characteristics for long-term stroke recurrence among patients with symptomatic intracranial atherosclerotic disease (ICAD).

**Methods:**

This cohort study included 132 patients with acute ischemic stroke (AIS) attributed to ICAD who were recruited between July 2017 and December 2020 and followed until stroke recurrence or December 2021. Plaque surface irregularity, degree of stenosis, plaque burden, remodeling ratio, enhancement ratio, and intraplaque hemorrhage were assessed with 3-dimensional high-resolution magnetic resonance vessel wall imaging (3D HR-MRI). Data were analyzed using Cox models, receiver operating characteristic (ROC) curves, and Kaplan–Meier survival analysis.

**Results:**

Of the 132 patients, during a median follow-up of 2.8 years, stroke recurrence occurred in 35 patients. The multivariable-adjusted hazard ratio (95% confidence interval) of stroke recurrence was 3.15 (1.34–7.42) per 10% increase in plaque burden and 2.17 (1.27–3.70) for enhancement ratio. The area under the curve (AUC) to predict stroke recurrence was 0.725 (95% CI 0.629–0.822) for plaque burden, 0.692 (95% CI 0.593–0.792) for enhancement ratio, and only 0.595 (95% CI 0.492–0.699) for the Essen stroke risk score. The Kaplan–Meier survival analysis further demonstrated significant differences in survival of free recurrent stroke between patients with plaque burden or enhancement ratio below and above the optimum cut-offs (both *p* < 0.001).

**Conclusion:**

Higher plaque burden and enhancement ratio are independent risk factors for long-term stroke recurrence among patients with symptomatic ICAD, and valuable imaging markers for predicting and stratifying risk of stroke recurrence.

**Clinical relevance statement:**

In patients with symptomatic ICAD, the results of this high-resolution magnetic resonance vessel wall imaging study have potential implications for optimal management of intracranial plaques and secondary prevention of stroke recurrence based on plaque burden and enhancement ratio.

**Key Points:**

*• Identification of intracranial plaque characteristics responsible for stroke recurrence is essential to preventing stroke recurrence in patients with symptomatic intracranial atherosclerotic disease.*

*• Higher plaque burden and enhancement ratio are independent risk factors for stroke recurrence.*

*• Plaque burden and enhancement ratio are valuable imaging markers in the prediction and stratification of the risk of stroke recurrence.*

**Supplementary Information:**

The online version contains supplementary material available at 10.1007/s00330-023-10278-y.

## Introduction

Intracranial atherosclerotic disease (ICAD) is a leading cause of ischemic stroke worldwide, especially in Asian populations [1]. Despite aggressive medical treatment, the annual risk of stroke recurrence is as high as 10–24% among patients with symptomatic ICAD [2]. Conventional imaging techniques, such as magnetic resonance angiography (MRA), computed tomography angiography (CTA), and digital subtraction angiography (DSA), could quantitatively assess the degree of lumen stenosis. However, the evaluation of lumen stenosis alone is not enough to reveal the severity of atherosclerotic lesions because it cannot characterize intracranial atherosclerotic plaques [3]. Therefore, to optimize treatment strategies and prevent stroke recurrence, there is a strong need to shift the evaluation from lumen stenosis to atherosclerotic plaque characteristics.

High-resolution magnetic resonance vessel wall imaging (HR-MRI) is a reliable and noninvasive technique to identify and characterize intracranial atherosclerotic plaques [4]. Most studies of intracranial plaques based on HR-MRI have been cross-sectional studies and have focused on discriminating plaque characteristics between symptomatic and asymptomatic patients [5, 6]. A recent meta-analysis concluded that plaque enhancement, positive remodeling, intraplaque hemorrhage, and surface irregularity are imaging characteristics of symptomatic intracranial plaques [7]. However, only a few studies have so far evaluated the value of plaque characteristics for predicting stroke recurrence in patients with symptomatic ICAD, and the results have been mixed. For example, a study from South Korea showed that intracranial plaque enhancement was associated with stroke recurrence and could predict stroke recurrence [8], whereas a study from China showed no significant association between plaque enhancement and stroke recurrence [9]. There is an urgent need to clarify the prognostic value of intracranial plaque characteristics in the assessment of stroke recurrence. Furthermore, most of the previous studies have used 2D HR-MRI to evaluate plaque characteristics mainly among patients with anterior circulation stroke, which may lead to inaccurate assessments of plaques due to the partial volume effect of thick slices. By contrast, 3D HR-MRI has higher resolution, thinner slices, better T1-weighted contrast, and advanced blood suppression techniques, which can accurately quantify plaque characteristics [10]. However, prospective cohort studies have not investigated the association of intracranial plaques quantitatively characterized using the 3D HR-MRI technique with long-term stroke recurrence among patients with symptomatic ICAD involving both anterior and posterior arteries.

Therefore, the purpose of this cohort study was to investigate the longitudinal associations of intracranial atherosclerotic plaque characteristics assessed using 3D HR-MRI technique with stroke recurrence in patients with symptomatic ICAD, and further to evaluate their predictive ability for stroke recurrence.

## Methods

### Patients

This study was approved by the ethical standards committees on human experimentation at Shandong Provincial Hospital, Shandong University. Written informed consent was obtained from all participants. This study was conducted in accordance with the ethical principles for medical research involving human subjects expressed in the Declaration of Helsinki.

In this prospective cohort study, patients were recruited at the Shandong Provincial Hospital who were hospitalized due to ischemic stroke and underwent 3D HR-MRI between July 2017 and December 2020. The patients underwent 3D HR-MRI when ICAD relevant to ischemic stroke was identified by CTA, MRA, or DSA. The inclusion criteria were as follows: (1) acute ischemic stroke (within four weeks from symptom onset to undertaking 3D HR-MRI); (2) age > 18 years; and (3) relevant intracranial artery plaques identified on 3D HR-MRI as the ischemic etiology. The exclusion criteria were as follows: (1) Over 50% stenosis of the ipsilateral extracranial artery detected by ultrasound, MRA, CTA, or DSA; (2) Potential source of cardiogenic embolism detected by an electrocardiogram (ECG), or echocardiography, such as atrial fibrillation, myocardial infarction within 3 weeks, and left atrial or ventricular thrombus on echocardiography; (3) presence of vasculopathy other than atherosclerosis, such as vasculitis, moyamoya disease, and dissection; (4) coagulation disorders; (5) unsatisfactory image quality of HR-MRI that could not be used to define the artery boundaries accurately for quantitative analysis; and (6) intracranial artery occlusion.

**Fig. 1:**
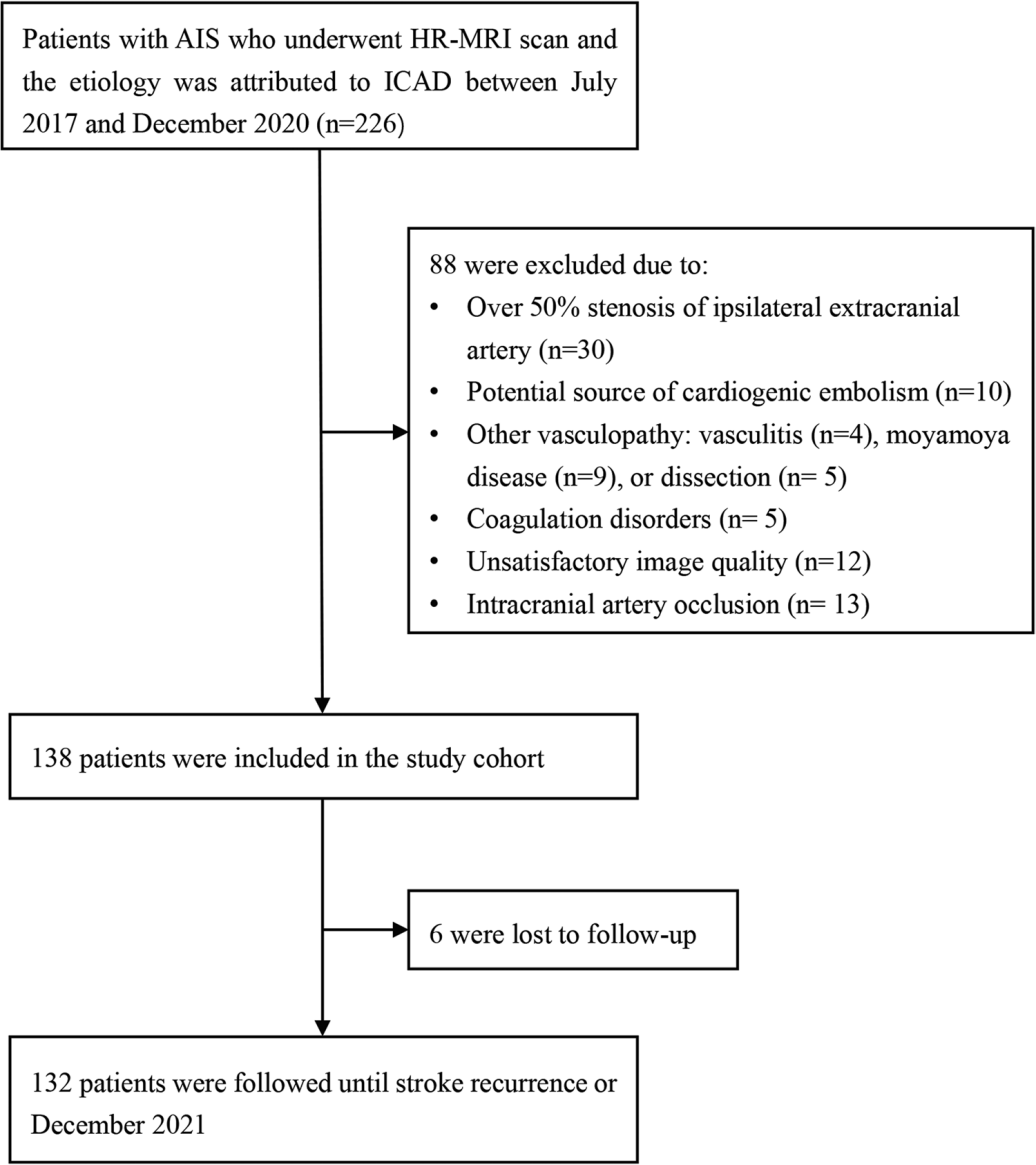
Flowchart of this study population. AIS: acute ischemic stroke; HR-MRI: high resolution magnetic resonance vessel wall imaging; ICAD: intracranial atherosclerotic disease

Demographic and clinical data, including age, gender, hypertension, diabetes mellitus, current smoking, blood lipid examination, coronary artery disease, previous stroke or transient ischemic attack (TIA), the National Institutes of Health Stroke Scale (NIHSS) score, and the Essen stroke risk score (ESRS), were recorded for each patient. All patients were treated according to guidelines of the American Heart Association/American Stroke Association for clinical management of cerebrovascular disorders [11, 12]. The flowchart of this study’s participants is shown in Fig. 1.

### High-resolution MR vessel wall imaging protocol

All eligible participants underwent brain MRI scans on a 3.0-T scanner (Achieve; Philips Medical Systems) with a sensitivity encoding (SENSE) parallel imaging head coil. The imaging protocols are listed in the supplemental materials.

### Image analysis

The evaluation of MR vessel wall imaging was independently conducted by two experienced neuroradiologists, using medical imaging viewer software (Extended MR WorkSpace, Philips Medical Systems). We assessed the following characteristics of culprit plaques: plaque surface irregularity, lumen stenosis, plaque burden, remodeling ratio, enhancement ratio, and intraplaque hemorrhage. The detailed definition and measurement of plaque characteristics are shown in the supplemental materials.

After two months of the initial evaluation, we randomly selected images of 30 patients to evaluate the inter-reader agreement in the assessments of plaque characteristics. There was an excellent inter-observer agreement for the identification and measurement of plaque characteristics.

### Outcome assessment

The main outcome of this study was the occurrence of a recurrent ischemic stroke in the same vascular territory during follow-up. The recurrent ischemic stroke was defined as a new neurological deficit or a sudden deterioration of a previous deficit that occurred over 21 days after the onset of the initial event and was attributable to a confirmed new cerebral infarct relevant to clinical symptoms in the same vascular territory as the initial event on brain imaging [13]. Further details are provided in the supplemental materials.

### Statistical analysis

We presented the mean (standard deviation) for continuous variables and frequency (%) for categorical variables. Comparisons between groups were conducted using t test for continuous variables and chi-square/Fisher test for categorical variables. Variables with *p* < 0.10 in the univariable Cox regression analysis were included in the multivariable Cox regression analysis to further identify independent factors associated with stroke recurrence. The receiver operating characteristic (ROC) curves were performed for variables independently associated with stroke recurrence, and area under the curve (AUC) was calculated. The optimal cut-offs of continuous variables were determined using ROC curves for Kaplan–Meier survival analysis. Kaplan–Meier survival analysis was performed to estimate the stroke recurrence-free rate. The log-rank test was used to compare the Kaplan–Meier survival curves. The inter-observer agreement of continuous variables was evaluated by intraclass correlation coefficient (ICC) using a two-way random model with absolute agreement. The inter-observer agreement of categorical variables was evaluated by Cohen’s kappa value. A value of ICC and kappa ≥ 0.81 indicates excellent agreement. Two-tailed *p* < 0.05 was considered statistically significant. IBM SPSS Statistics for Windows, Version 26, released in 2019 (IBM Corp.) was used for all statistical analyses.

## Results

### Incidence of recurrent stroke

The mean age of the 132 patients was 59.83 (SD = 11.06) years and 46.2% were female. The culprit vessels assessed using the 3D HR-MRI included 15 intracranial internal carotid arteries, 55 middle cerebral arteries, 14 vertebral arteries, and 48 basilar arteries. During a total of 346.6 person-years of follow-up (median per person 2.8 years; interquartile range: 1.3–3.8 years), recurrent stroke occurred in 35 patients. The incidence of recurrent stroke was 10.1 per 100 person-years.

### Clinical and plaque characteristics of patients

The proportion of hypertension and diabetes mellitus was significantly higher in patients with stroke recurrence than in patients without (*p* = 0.030 and 0.047, respectively). No significant differences were observed in other clinical features (Table 1).

**Table 1:** Clinical characteristics of patients with symptomatic ICAD in the total sample and by stroke recurrence at follow-up

Characteristics	Total sample (*N* = 132)	Stroke recurrence at follow-up		
		Yes (*N* = 35)	No (*N* = 97)	*p* value
Age (year)	59.83 (11.06)	59.34 (9.87)	60.01 (11.50)	0.761
Male, *n* (%)	71 (53.8)	17 (48.6)	54 (55.7)	0.470
Smoking, *n* (%)	23 (17.4)	5 (14.3)	18 (18.6)	0.568
Hypertension, *n* (%)	90 (68.2)	29 (82.9)	61 (62.9)	0.030
Diabetes mellitus, *n* (%)	46 (34.8)	17 (48.6)	29 (29.9)	0.047
Previous stroke or TIA, *n* (%)	35 (26.5)	10 (28.6)	25 (25.8)	0.748
Coronary artery disease, *n* (%)	18 (13.6)	4 (11.4)	14 (14.4)	0.780
Total cholesterol (mmol/l)	4.17 (1.03)	4.12 (1.20)	4.19 (0.97)	0.754
HDL (mmol/l)	1.14 (0.26)	1.09 (0.23)	1.15 (0.26)	0.240
LDL (mmol/l)	2.50 (0.73)	2.47 (0.82)	2.51 (0.70)	0.762
Triglyceride (mmol/l)	1.50 (0.86)	1.68 (1.34)	1.43 (0.59)	0.298
NIHSS score	3.33 (1.83)	3.31 (1.92)	3.34 (1.81)	0.943
ESRS	2.05 (1.21)	2.31 (1.02)	1.96 (1.27)	0.138
Anterior circulation territory, n (%)	70 (53.0)	19 (54.3)	51 (52.6)	0.862
Interval from symptom onset to imaging (days)	11.14 (8.41)	11.17 (8.81)	11.13 (8.31)	0.982

Data were mean (standard deviation), unless otherwise specified

*HDL* high-density lipoprotein cholesterol, *LDL* low-density lipoprotein cholesterol, *NIHSS* National Institutes of Health Stroke Scale, *ESRS* Essen stroke risk score, *TIA* transient ischemic attack

**Fig. 2:**
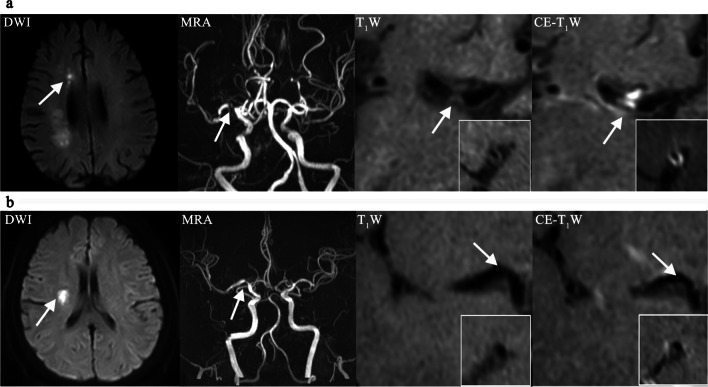
Example images of patients with and without stroke recurrence. **a**. A patient with stroke recurrence. DWI shows infarct in the right corona radiata (arrow). MRA shows severe stenosis on the M1 segment of right MCA (arrow). Pre- (T_1_W) and post-contrast (CE-T_1_W) HR-MRI detect a plaque (arrow) with higher plaque burden (89.8%) and enhancement ratio (0.84). **b**. A patient without stroke recurrence. DWI shows infarct in the right corona radiata (arrow). MRA shows moderate stenosis on the M1 segment of right MCA (arrow). Pre- (T_1_W) and post-contrast (CE-T_1_W) HR-MRI detect a plaque (arrow) with smaller plaque burden (81.5%) and enhancement ratio (0.27). DWI: diffusion weighted imaging; MRA: magnetic resonance angiography; MCA: middle cerebral artery

Patients with stroke recurrence had significantly higher plaque burden (*p* < 0.001), enhancement ratio (*p* = 0.001), and degree of stenosis (*p* = 0.003) than those without stroke recurrence, whereas the two groups had no significant differences in plaque surface irregularity (*p* = 0.501), intraplaque hemorrhage (*p* = 0.438), and remodeling ratio (*p* = 0.851) (Table 2). Example images of patients with and without stroke recurrence are shown in Fig. 2.

**Table 2:** Plaque characteristics of patients with symptomatic ICAD in the total sample and by stroke recurrence at follow-up

Characteristics	Total sample (*N* = 132)	Stroke recurrence at follow-up		
		Yes (*N* = 35)	No (*N* = 97)	*p* value
Surface irregularity, *n* (%)	43 (32.6)	13 (37.1)	30 (30.9)	0.501
Intraplaque hemorrhage, *n* (%)	14 (10.6)	2 (5.7)	12 (12.4)	0.438
Stenosis degree (%)	59.7 (21.2)	67.7 (16.4)	56.8 (22.1)	0.003
Plaque burden (%)	87.0 (6.7)	90.5 (5.2)	85.7 (6.7)	< 0.001
Remodeling ratio	1.04 (0.34)	1.03 (0.34)	1.04 (0.33)	0.851
Enhancement ratio	0.66 (0.54)	0.91 (0.63)	0.57 (0.47)	0.001

Data were mean (standard deviation), unless otherwise specified

### Association between plaque characteristics and stroke recurrence

Univariable and multivariable Cox regression analyses were used to detect factors associated with stroke recurrence. Hypertension, diabetes mellitus, stenosis degree, plaque burden, and enhancement ratio were associated with stroke recurrence at the *p* < 0.10 level in the univariable analysis, and they were included in the multivariable Cox regression model (Table 3). Multivariable Cox regression analysis demonstrated that plaque burden (per 10% increase, HR = 3.15; 95% CI 1.34–7.42; *p* = 0.009) and enhancement ratio (HR = 2.17; 95% CI 1.27–3.70; *p* = 0.004) were independently associated with stroke recurrence (Table 3). When stenosis degree was graded as mild-moderate (< 70%) and severe (≥ 70%), greater plaque burden (HR = 4.03, per 10% increase; 95% CI 1.73–9.38; *p* = 0.001) and higher enhancement ratio (HR = 2.32; 95% CI 1.35–3.99; *p* = 0.002) remained independently associated with an increased risk of stroke recurrence.

**Table 3:** Hazard ratio (95% confidence interval) of stroke recurrence associated with various risk factors from Cox regression models

Risk factors	Univariable model		Multivariable model	
	HR (95%CI)	*p* value	HR (95%CI)	*p* value
Hypertension	2.47 (1.02–5.94)	0.044	2.00 (0.82–4.88)	0.129
Diabetes mellitus	1.94 (1.00–3.76)	0.051	1.49 (0.74–3.01)	0.267
Stenosis degree^a^	1.26 (1.05–1.53)	0.015	0.99 (0.79–1.25)	0.947
Plaque burden^a^	3.18 (1.65–6.10)	0.001	3.15 (1.34–7.42)	0.009
Enhancement ratio	2.63 (1.59–4.34)	< 0.001	2.17 (1.27–3.70)	0.004

^a^ HR and 95% confidence interval were estimated based on every 10% increase

*HR* hazard ratio, *CI* confidence interval

**Fig. 3:**
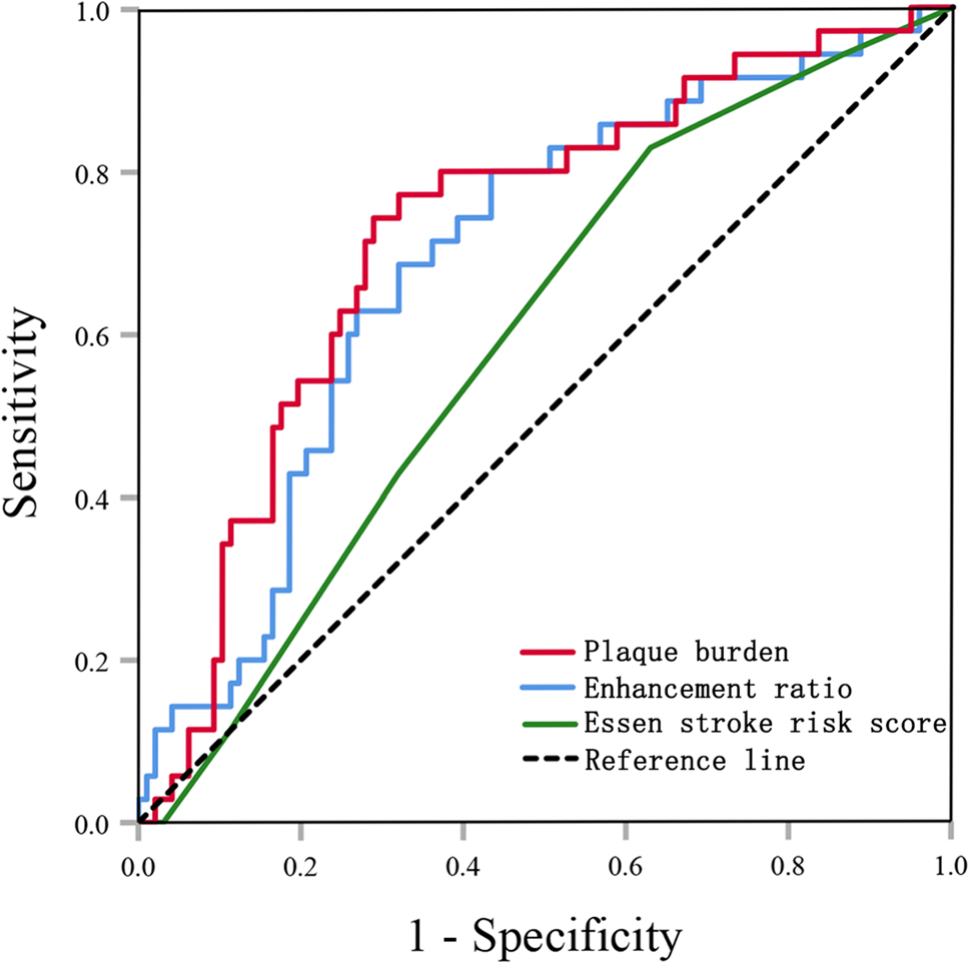
Receiver operating characteristic (ROC) curves in predicting stroke recurrence. Plaque burden: The area under the curve (AUC) = 0.725 (95% CI 0.629–0.822), cut-off = 89.2%. Enhancement ratio: AUC = 0.692 (95% CI 0.593–0.792), cut-off = 0.50. Essen stroke risk score (ESRS): AUC = 0.595 (95% CI 0.492–0.699)

The ROC curves were used to evaluate the predictive ability of plaque burden and enhancement ratio for stroke recurrence (Fig. 3). The AUC was 0.725 (95% CI 0.629–0.822) for plaque burden and 0.692 (95% CI 0.593–0.792) for enhancement ratio. The AUC for the Essen stroke risk score (ESRS) was 0.595 (95% CI 0.492–0.699). Predictive parameters of plaque burden, enhancement ratio, and ESRS for stroke recurrence are shown in the supplemental materials. The optimal cut-offs of plaque burden and enhancement ratio for predicting stroke recurrence were 89.2% and 0.50, respectively.

**Fig. 4:**
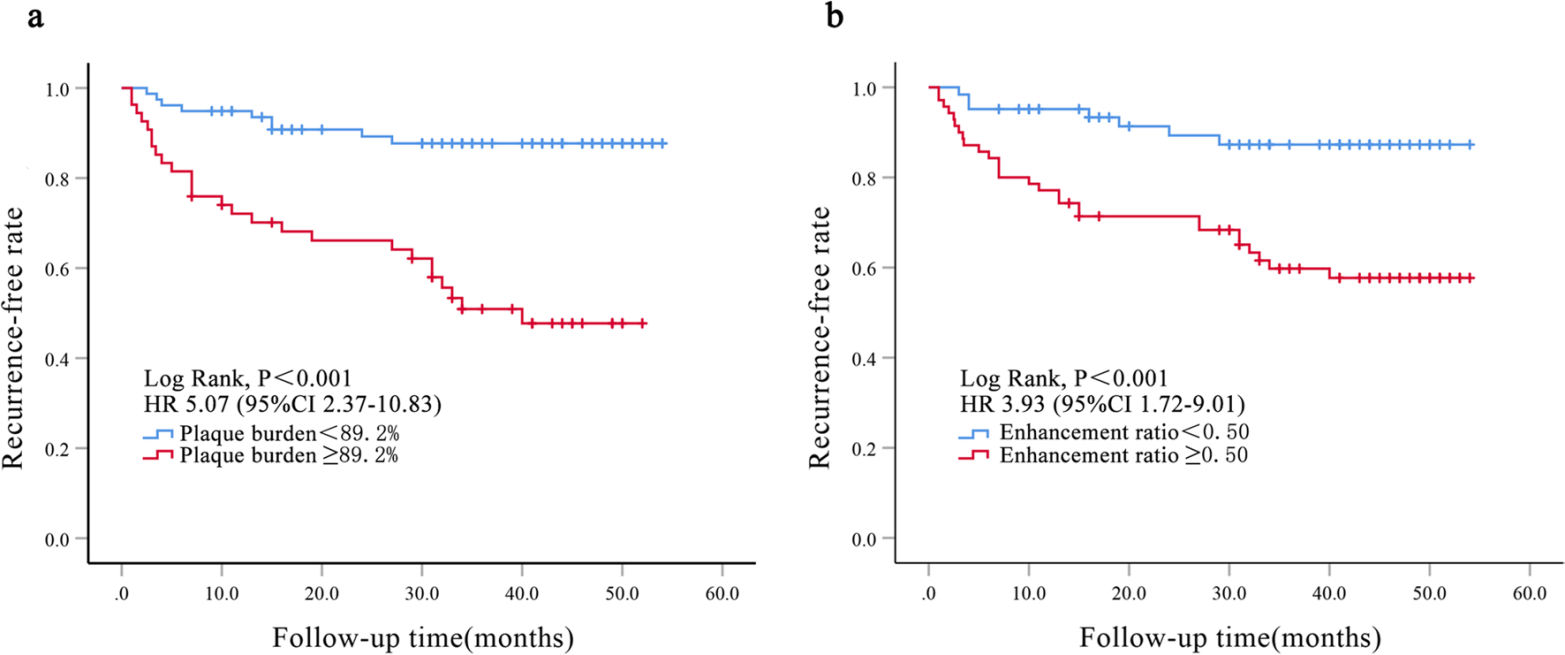
Kaplan–Meier curves of survival free of stroke recurrence stratified by plaque burden (**a**) and enhancement ratio (**b**). There was a significant difference between the recurrence risk stratified by the optimum cut-off of plaque burden (89.2%) or enhancement ratio (0.50) (both *p* < 0.001)

Furthermore, we used Kaplan–Meier survival analysis to illustrate the risk of stroke recurrence by the optimal cut-off of plaque burden (89.2%) and enhancement ratio (0.50). The risk of stroke recurrence was significantly higher in patients with plaque burden ≥ 89.2% than those with plaque burden < 89.2% (HR 5.07; 95% CI 2.37–10.83; *p* < 0.001; Fig. 4a), and in patients with enhancement ratio ≥ 0.50 than those with enhancement ratio < 0.50 (HR 3.93; 95% CI 1.72–9.01; *p* < 0.001; Fig. 4b).

To assess whether the evolvement of plaque features over time and the patients’ compliance to treatments could affect the main results, we performed sensitivity analysis by examining the association between the plaque features and stroke recurrence at the 1-year follow-up. Recurrent stroke occurred in 19 patients at the 1-year follow-up. Cox regression analysis suggested that plaque burden (per 10% increase, HR = 2.75; 95% CI 1.09–6.93; *p* = 0.032) and enhancement ratio (HR = 2.62; 95% CI 1.36–5.04; *p* = 0.004) independently associated with stroke recurrence and that no other examined plaque characteristics were significantly associated with stroke recurrence.

## Discussion

In this cohort study, we found that higher plaque burden and enhancement ratio, quantified using 3D HR-MRI, were independent risk factors associated with long-term stroke recurrence. Plaque burden and enhancement ratio, but not ESRS, had good predictive ability for stroke recurrence. Furthermore, patients with plaque burden ≥ 89.2% or enhancement ratio ≥ 0.50 had over 4- and twofold increased risk of stroke recurrence, respectively.

Plaque burden could accurately indicate the severity of atherosclerosis beyond lumen stenosis. It has been demonstrated that a larger plaque burden in the coronary arteries is a risk factor for major adverse cardiovascular events and a plaque burden ≥ 70% can predict future cardiovascular events [14]. Some previous studies also showed that a larger plaque burden was detected in culprit plaques in the middle cerebral artery compared with non-culprit plaques, and plaque burden was a better indicator in discriminating between culprit and non-culprit plaques [15, 16]. Larger plaque burden was significantly associated with the severity of acute ischemic stroke in the middle cerebral artery and internal carotid artery [17]. However, very few cohort studies have investigated the association between intracranial plaque burden and subsequent risk of stroke recurrence. Although two previous cross-sectional studies showed that plaque burden was independently associated with stroke recurrence [18, 19], the findings from cross-sectional studies cannot provide evidence for a causal relationship between plaque burden with stroke recurrence in patients with symptomatic ICAD. Our relatively large-scale prospective cohort study used 3D HR-MRI with higher resolution and thinner slices compared with the commonly used 2D high-resolution sequences, which can improve the accuracy of quantitative measurements for plaque burden and other characteristics. Therefore, this study provides direct evidence suggesting that plaque burden is an independent risk factor for stroke recurrence and may be used to predict and stratify the risk of stroke recurrence in Chinese patients with symptomatic ICAD.

Evidence from studies of the extracranial carotid arteries showed that plaque gadolinium enhancement was correspondent to histological markers of inflammation and neovascularization, and it was identified as an important marker of plaque vulnerability [20]. Plaque enhancement may be related to the endothelial dysfunction of intraplaque microvessels, which facilitates the delivery and accumulation of gadolinium into plaque [21]. Because intracranial arteries are relatively inaccessible, the histopathology of intracranial plaque enhancement has not been validated yet, and much of the current knowledge of histopathology of intracranial plaque enhancement has been derived from studies of extracranial atherosclerosis [22]. Although some studies indicated that vasa vasorum might develop in the proximal segments of intracranial arteries in the process of atherosclerosis [23], intracranial arteries have distinct basic structures from similar size extracranial arteries, including denser internal elastic lamina, thinner media, less abundant adventitia without external elastic lamina, and paucity of vasa vasorum, which may partly contribute to the difference in atherosclerosis between intracranial and extracranial arteries [22]. Therefore, the extrapolation of histopathology of plaque enhancement from extracranial atherosclerosis to intracranial atherosclerosis needs to be further verified.

In spite of lack of the validation of histopathology, recent studies have revealed the clinical significance of intracranial plaque enhancement [7, 24]. However, to date, follow-up data on the association between intracranial plaque enhancement and stroke recurrence are limited and inconsistent. Previously, two short-term (1–1.5 years) follow-up studies from Korea and China showed that qualitative plaque enhancement was independently associated with stroke recurrence in patients with intracranial atherosclerosis, even after adjustment for collateral status and infarct pattern [8, 25]. By contrast, the 1-year follow-up study in China did not find an association between qualitative plaque enhancement and stroke recurrence in symptomatic ICAD patients [9]. This inconsistency may be partly due to the use of visual qualitative assessment for plaque enhancement. The subjective interpretation of plaque signals by raters may affect the accuracy of plaque enhancement assessment. Our study used an enhancement ratio to quantitatively assess plaque enhancement, which could be more reliable compared with visually qualitative assessment [26]. A recent cross-sectional study showed that a higher enhancement ratio of symptomatic intracranial plaques was independently associated with recurrent acute stroke in patients with first-time acute stroke [19]. Our cohort study further showed that a higher enhancement ratio was an independent risk factor responsible for stroke recurrence and had the prognostic value for predicting stroke recurrence in patients with symptomatic ICAD.

Although ESRS is a well-known tool for predicting stroke recurrence based on clinical variables, the ESRS may not have sufficient discriminatory ability. For example, the AUC for the ESRS was 0.60 in REduction of Atherothrombosis for Continued Health (REACH) registry study, 0.61 in the Systemic Risk Score Evaluation in Ischaemic Stroke Patients (SCALA) study, and 0.60 in China National Stroke Registry (CNSR) study [27–29]. The relatively low predictive ability of ESRS indicated that ESRS may not reflect the critical factors that predispose to stroke recurrence. Our study showed that the predictive ability of plaque burden and enhancement ratio for stroke recurrence was much better than ESRS (AUC 0.725 and 0.692 vs. 0.595). Furthermore, patients with plaque burden ≥ 89.2% or enhancement ratio ≥ 0.50 had a substantial risk of stroke recurrence. Therefore, our cohort study supported that plaque burden and enhancement ratio were promising imaging markers in the prediction and risk stratification of stroke recurrence in symptomatic ICAD patients. Both plaque burden and enhancement ratio seem suitable for application in clinical practice to increase awareness of recurrent stroke risk and have the potential for optimizing secondary prevention strategies in symptomatic ICAD patients.

The degree of artery stenosis has been identified as a risk factor for stroke recurrence [1], whereas our study showed no significant association between the degree of lumen stenosis and stroke recurrence. There is increasing evidence suggesting the limitation of stenosis degree in determining the risk of stroke recurrence in patients with symptomatic ICAD. In the Warfarin versus Aspirin for Symptomatic Intracranial Disease (WASID) trial, the role of stenosis degree in predicting stroke recurrence was superseded by collateral flow [30]. In the Stroke Outcomes and Neuroimaging of Intracranial Atherosclerosis (SONIA) study, hemodynamics was identified as an independent predictor for stroke recurrence, whereas stenosis degree was not found to be associated with stroke recurrence in patients with symptomatic ICAD [31]. Our study further emphasizes the importance of plaque characteristics rather than lumen stenosis in determining the risk of stroke recurrence in patients with symptomatic ICAD.

The prognostic value of plaque burden and enhancement ratio for stroke recurrence among patients with symptomatic ICAD has been rarely evaluated in prospective cohort studies with a long-term follow-up period. Furthermore, our study used 3D high-resolution sequences to assess plaque parameters, which could provide more accurate quantitative measurements of plaque characteristics than those of qualitative 2D high-resolution sequences [10]. Finally, this cohort study included patients with symptomatic ICAD involving both anterior and posterior arteries. Our findings may be more generalizable compared with those studies of only the middle cerebral artery or basilar artery.

Our study also has limitations. Firstly, this is a single-center study with a relatively small sample size, which limits the power to show the association between other plaque characteristics with stroke recurrence. Multi-center studies with a larger sample size are needed in the future. Secondly, we did not evaluate collateral flow and hemodynamics that may play a role in stroke recurrence, which might partly contribute to the lack of association between severe stenosis and stroke recurrence. Further studies incorporating plaque characteristics, collateral flow, and hemodynamics may provide more information for assessing the risk of stroke recurrence. Lastly, intracranial atherosclerosis is a dynamic process showing both progression and regression over time [32, 33]. We did not monitor the evolution of plaque characteristics during the follow-up period.

In conclusion, higher plaque burden and enhancement ratio were independent risk factors for long-term stroke recurrence in patients with symptomatic ICAD. Plaque burden and enhancement ratio are valuable intracranial plaque parameters for predicting the risk of recurrent stroke and for risk stratification of stroke patients with symptomatic ICAD. These findings have potential implications for optimal management of intracranial plaques and secondary prevention of stroke recurrence in patients with symptomatic ICAD.

### Supplementary Information

Below is the link to the electronic supplementary material.

Supplementary file1 (PDF 173 KB)

## Abbreviations

3D T1-VISTA: T1 weighted imaging with 3D variable refocusing flip angle volume isotropic turbo spin-echo acquisition
AIS: Acute ischemic stroke
AUC: Area under the curve
CI: Confidence interval
DSA: Digital subtraction angiography
ESRS: Essen stroke risk score
HDL: High-density lipoprotein cholesterol
HR: Hazard ratio
HR-MRI: High-resolution magnetic resonance vessel wall imaging
ICAD: Intracranial atherosclerotic disease
ICC: Intraclass correlation coefficient
LA: Lumen area
LDL: Low-density lipoprotein cholesterol
MRA: Magnetic resonance angiography
NIHSS: National Institutes of Health Stroke Scale
ROC: Receiver operating characteristic
TC: Total cholesterol
TG: Triglyceride
VA: Vessel area
WA: Wall area

## Declarations

### Guarantor

The scientific guarantor of this publication is Qinjian Sun, MD.

### Statistics and biometry

No complex statistical methods were necessary for this paper.

### Study subjects or cohorts overlap

No study subjects or cohorts have been previously reported.

### Methodology

• prospective

• observational

• performed at one institution
