# Supplementary material for: Association of plaque characteristics with long-term stroke recurrence in patients with intracranial atherosclerotic disease: a 3D high-resolution MRI-based cohort study
Source: Eur Radiol. 2023 Oct 23;34(5):3022–31. doi: 10.1007/s00330-023-10278-y (PMC11126465; doi:10.1007/s00330-023-10278-y)
Supplement: Supplementary file 1 — Supplementary file1 (PDF 173 KB) [file 330_2023_10278_MOESM1_ESM.pdf]

Association of plaque characteristics with long-term stroke recurrence in patients with intracranial atherosclerotic disease: A 3D high-resolution MRI-based cohort study

Electronic Supplementary Material

Supplemental Methods

High-resolution MR vessel wall imaging protocol

All eligible participants underwent brain MRI scans on a 3.0T scanner (Achieve; Philips Medical Systems) with a sensitivity encoding (SENSE) parallel imaging head coil. MRA was obtained in an axial plane with the following parameters: repetition time/echo time (TR/TE), 27/6.9 ms; flip angle 20°; field-of-view (FOV) 24 cm × 16 cm; matrix size 320 × 256; slice thickness 1.6 mm. T1 weighted imaging with 3D variable refocusing flip angle volume isotropic turbo spin-echo acquisition (3D T1-VISTA) was performed, using the following scan parameters: repetition time (TR)/echo time (TE), 800/18 ms; echo train length (ETL), 16; field of view (FOV), 200 mm×180 mm×40 mm; slice thickness, 0.3 mm; matrix, 332×302 (yielding a 0.3 mm ×0.3 mm ×0.3 mm isotropic spatial resolution); number of averages, 1-2; parallel imaging (SENSE) factor, 2 (along the phase-encoding direction); and scan time, 378s. Sensitized flow compensation was applied to suppress intraluminal blood signals originating from the arteries. Low refocusing flip angles (90°) were used to increase flow-void effects and decrease image. The contrast enhanced T1-VISTA parameters were the same with those of pre-contrast T1-VISTA.

Image analysis

The evaluation of MR vessel wall imaging was independently conducted by two experienced neuroradiologists, using medical imaging viewer software (Extended MR WorkSpace, Philips Medical Systems). Image quality was graded using a four-point scale (1, poor; 2, marginal; 3,

good; and 4, excellent) based on the overall signal-to-noise ratio and the clarity of the vessel wall boundaries [1], and the images with image quality ≥3 were qualified for analysis. Atherosclerotic plaques on MR vessel wall imaging were identified using a previously reported definition, i.e., the presence of focal wall thickening with or without significant luminal stenosis[2]. A plaque was considered a culprit plaque when it was (a) the only lesion within the vascular territory of the stroke or (b) the most stenotic lesion when multiple plaques were present within the same vascular territory of the stroke [2].

The culprit plaque characteristics, including plaque surface irregularity, lumen stenosis, plaque burden, remodeling ratio, enhancement ratio and intraplaque hemorrhage, were analyzed. The normal vessel segments proximal to the culprit plaques were assessed as reference values. Plaque surface irregularity was defined as a discontinuity of the plaque juxtaluminal surface, and regularity defined as smooth inner wall [3]. The vessel area (VA) and lumen area (LA) were automatically calculated by manually tracing the outer vessel and lumen contours. The wall area (WA) was the difference between vessel area and lumen area. The stenosis degree was calculated as: (1-lesion lumen area/reference lumen area) ×100% [4]. Plaque burden was calculated as: wall area (WA)/vessel area (VA) ×100% [5]. The schematic diagram regarding the measurement of the plaque burden is shown in Figure S1. Remodeling ratio was calculated as the ratio of the lesion vessel area to the reference vessel area [4]. Enhancement ratio was measured at the slice of greatest enhancement, using adjacent gray matter (in a region of ~ 15 mm^2) to normalize signal intensity. Enhancement ratio was calculated as: [signal of plaque (post-contrast)/signal of gray matter (post-contrast)-signal of plaque (pre-contrast)/signal of gray matter (pre-contrast)]/[signal of plaque (pre-contrast)/signal of gray matter (pre-contrast)][6]. Intraplaque hemorrhage was defined as >150% signal relative to nearby muscles on pre-contrast T1-weighted images [6].

After two months of the initial evaluation, we randomly selected images of 30 patients to

evaluate the inter-reader agreement in the assessments of plaque characteristics. There was excellent inter-observer agreement for the identification and measurement of plaque characteristics. The intraclass correlation coefficient (ICC) values were 0.951 (95% CI 0.858-0.980) for degree of stenosis, 0.939 (95% CI 0.821-0.975) for plaque burden, 0.952 (95% CI 0.881-0.979) for enhancement ratio, and 0.933 (95% CI 0.866-0.967) for remodeling ratio. The Kappa values were 0.83 for surface irregularity, and 1.0 for intraplaque hemorrhage.

Outcome assessment

The main outcome of this study was occurrence of a recurrent ischemic stroke in the same vascular territory during follow-up. The recurrent stroke was defined as a new neurological deficit or a sudden deterioration of a previous deficit which occurred over 21 days after the onset of the initial event and was attributable to a confirmed new cerebral infarct relevant to clinical symptoms in the same vascular territory as the initial event on brain imaging [7]. Patients were followed up via face-to-face or telephone interviews until occurrence of recurrent stroke or 31 December 2021, whichever came first. The recurrent stroke was diagnosed and confirmed by the routine head MRI scan, including DWI. The detailed information about the recurrence of stroke, risk factors and current medical treatment were recorded.

Table S1. Predictive parameters of plaque burden, enhancement ratio, and ESRS for stroke recurrence

Predictive parameters	AUC (95% CI)	Sensitivity	Specificity	Youden Index
Plaque burden	0.725 (0.629-0.822)	0.74	0.71	0.45
Enhancement ratio	0.692 (0.593-0.792)	0.80	0.57	0.37
ESRS	0.595 (0.492-0.699)	0.83	0.37	0.20

ESRS: Essen stroke risk score; AUC: Area under the curve.

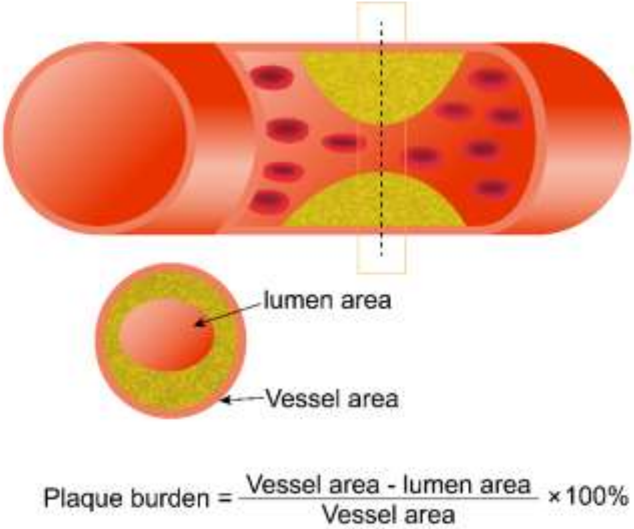

Figure S1. The schematic diagram regarding measurement of the plaque burden.

References

1 Lu M, Peng P, Cui Y et al (2018) Association of Progression of Carotid Artery Wall Volume and Recurrent Transient Ischemic Attack or Stroke: A Magnetic Resonance Imaging Study. Stroke 49:614-620.

2 Qiao Y, Zeiler SR, Mirbagheri S et al (2014) Intracranial plaque enhancement in patients with cerebrovascular events on high-spatial-resolution MR images. Radiology 271:534-542.6

3 Wu F, Song H, Ma Q et al (2018) Hyperintense Plaque on Intracranial Vessel Wall Magnetic Resonance Imaging as a Predictor of Artery-to-Artery Embolic Infarction. Stroke 49:905-911.

4 Chung JW, Hwang J, Lee MJ, Cha J, Bang OY (2016) Previous Statin Use and High-Resolution Magnetic Resonance Imaging Characteristics of Intracranial Atherosclerotic Plaque: The Intensive Statin Treatment in Acute Ischemic Stroke Patients With Intracranial Atherosclerosis Study. Stroke 47:1789-1796.

5 Wang M, Wu F, Yang Y et al (2018) Quantitative assessment of symptomatic intracranial atherosclerosis and lenticulostriate arteries in recent stroke patients using whole-brain high-resolution cardiovascular magnetic resonance imaging. J Cardiovasc Magn Reson 20:35.

6 Zhu C, Tian X, Degnan AJ et al (2018) Clinical Significance of Intraplaque Hemorrhage in Low- and High-Grade Basilar Artery Stenosis on High-Resolution MRI. AJNR Am J Neuroradiol 39:1286-1292.

7 Coull AJ, Rothwell PM (2004) Underestimation of the early risk of recurrent stroke: evidence of the need for a standard definition. Stroke 35:1925-1929.
